# Unequal Distribution of Overweight Adolescents in Immigrant-Rich Areas: Analysis of Disparities among Public and Private School Students in Shanghai, China

**DOI:** 10.3390/ijerph14030252

**Published:** 2017-03-02

**Authors:** Jianwei Shi, Duxun Tan, Huilin Xie, Beilei Yang, Rui Liu, Dehua Yu, Yuan Lu, Bing Mei, Zhaoxin Wang

**Affiliations:** 1 Yangpu Hospital, Tongji University School of Medicine, Shanghai 200090, China; shijianwei_amy@126.com (J.S.); ydh1404@sina.com (D.Y.); lussy@126.com (Y.L.); 2 School of Medicine, Tongji University, Shanghai 200092, China; 3 The Fifth Affiliated Hospital of Southern Medical University, Guangzhou 510900, China; tanxynf@163.com (D.T.); jchtj2012@163.com (H.X.); 4 College of Economic and Management, Tongji University, Shanghai 200092, China; yblei@vip.163.com; 5 Shanghai Tenth People’s Hospital, Tongji University School of Medicine, Shanghai 200072, China; liurui@tongji.edu.cn; 6 Department of Emergency, Shanghai Changhai Hospital, Second Military Medical University, Shanghai 200433, China

**Keywords:** adolescent overweight, immigrant students, education, developing regions

## Abstract

Accelerated urbanization and rising immigration to the big cities in China has resulted in education policies that produce disparate treatment of immigrant and non-immigrant students. The two types of students frequently wind up in different types of junior high schools. However, there is little research on whether disparities exist between students in public and private schools with regard to overweight. This study aims to address this gap through a comparison of the overweight status of junior high school students in public and private schools in Shanghai and explore the possible reasons for the observed differences. Students from two public and two private junior high schools were measured. In order to determine what factors might shape overweight among adolescents. Logistic regression analysis was used to assess associations between overweight and personal characteristics, birth-related factors, levels of physical activity, diet, family socioeconomic status and school environment. Students in private schools proved more likely to be overweight (15.20%, *p* < 0.05) than public school students (10.18%). Similarly, gender, breastfeeding, parental care and number of classes excluding physical education per day were found to be significant factors. However, private school students were also influenced by gestational age (yes/no: OR = 4.50, *p* < 0.001), frequency of snacks (sometimes/often: OR = 0.53, *p* < 0.01) and family income (¥6001–12,000/below ¥6000: OR = 3.27, *p* < 0.05). Time for lunch was the sole risk factor for public school students in the study (*p* < 0.05). To reduce the unequal distribution of overweight students between the two types of schools, interventions that consider different multiple risk factors should be implemented.

## 1. Introduction

Rapid urbanization and changes in living standards in developing countries such as China, are accompanied by increased adolescent overweight. This development has attracted a growing concern from both the government and the public. A growing body of research indicates that the causes of adolescent obesity involves a variety of factors, including children’s personal characteristics (e.g., age, gender, ethnicity), maternal healthy behaviors (i.e., breastfeeding, maternal smoking behavior), children’s physical activity and sedentary behaviours, diet (i.e., food nutrition, food access), family socioeconomic position (e.g., parents’ education, income and occupation), and school environment [1,2,3,4,5,6,7,8,9,10,11,12]. 

In recent years, studies on excessive weight among adolescents have focused primarily on family and school environmental determinants; adolescent overweight results from inequities produced by the environment with regard to children’s growth. Much research recommends that special attention be given to vulnerable populations [4,13,14]. For example, Nicosia et al. (2016) propose that substantial disparities in BMI persisted between U.S. Black and White girls, and that the Black girls should be provided additional care [12]. With rapid urbanization in China, big cities are attracting more immigrants. In late 2015, governmental statistics showed that the number of the long-term residents in Shanghai was 14.33 million while the migrant population reached 9.81 million [15]. In order to control the dramatic increase in migration in these highly-developed cities, strict registration policy was implemented from the early 2000s for migrants and only the elite immigrants may access the new census register of local identification. In this case, only those students whose parents enjoy registered residence in the city may be accepted by good public elementary and secondary schools [16]. This situation is very different from many western countries. In China, due to a sufficient financial allocation for public education, public schools have more advantages to recruit good teachers and enjoy adequate resources. In light of the challenge to provide education for incoming migrant students, private schools obtained a privileged market position. Consequently, many of them have achieved great development. In order to obtain a decent education for their children, immigrant parents often must choose good private schools. The very different environments in public and private schools may contribute to disparities in excessive weight among the students.

To date, very few studies have focused on this phenomenon and associated obesity with possible factors related to students’ health. Liu et al. (2016) observed that there existed significant differences in childhood obesity among primary school children in private and public schools in Guangzhou [2]. However, their analysis lacked the systematic analysis about the differences of overweight students in private and public schools; nor did they explore the reasons for the difference. A study on the Gambia adolescent population noted that private school students were more likely to be overweight than public school students in urban Gambia due to malnutrition [17]. However, the situation in China is more complex and few researchers have fully explored and identified the possible factors that contribute to the differences in excess weight among students in public and private schools. 

In this study, we hypothesized that there would be a significant difference in the numbers of overweight students between public and private schools in cities with large numbers of immigrants, and we hypothesize that students’ personal characteristics, activity and diet, socio-economic factors, and school environment may influence this phenomenon. For this reason, the purpose of this study was (1) to determine whether there was any significant disparity in the number of overweight students between public and private schools in Shanghai, which has a large immigrant population; (2) to examine the possible factors that contribute to it, including personal characteristics, such as birth-related factors, student’ activities and diet, socio-economic factors, as well as the school environment. Finally, recommendations were made to improve the health status of vulnerable students impacted by social problems that resulted from immigrants in China and other developing regions. We think that this study will provide information to help students from different types of schools in China’s immigrant-rich cities and in other developing countries who may be in the same situation to better manage their fitness. 

## 2. Methods

### 2.1. Sample

A multi-stage stratified cluster random sampling method was used to obtain a representative sample of secondary students in two districts of Shanghai. To make the sample comparable, public and private schools with the high rankings in their local districts were included. The rankings were based on the enrolment rate, academic quality, hardware equipment, and other factors. Each year, it is assessed and released by the Education Bureau. Using a random number generator, two public and two private junior high schools were chosen. Within each school, two classes per year (from the 7th and 8th grades) were randomly selected and all students were invited to participate. Anthropometric measurements were taken and participants completed a survey with questions about their physical activity, diet, and school environment. The student’s parents were asked to attend the parent questionnaire on student’s birth-related factors and socioeconomic status. Both parts of the study were conducted during the period of April 2014 to June 2014. A total of 1012 students and their parents from these schools were invited to participate in the surveys. Finally, 989 valid questionnaires were collected from the students and 963 from those students’ parents. In total, there were 952 valid surveys, among which 452 were from the public schools and 500 were from the private schools.

### 2.2. Measurements

#### 2.2.1. Adolescent Overweight 

Children were classified as either overweight or not overweight. Overweight was defined according to the International Obesity Taskforce (IOTF) body mass index (kg/m^2^ cut-points) [18]. In this study, anthropometric measurements were taken by trained research staff. Students were weighed without shoes or outdoor clothing and weights were recorded to the nearest 0.1 kg by using an electronic scale (HCS-200-RT, Liheng Instrument Co. Ltd., Shanghai, China). Heights also were recorded to the nearest millimetre by using this scale. Height and weight were measured twice to minimize error.

#### 2.2.2. Personal Characteristics

We included gender and age as demographic variables that may impact adolescent overweight [3].

#### 2.2.3. Factors Related to Early Infancy

Birth-related factors consisted of birth weight (overweight: ≥4 kg, non-overweight: <4 kg) [12], gestational age (≥42 weeks, <42 weeks) [19], and whether or not the child was breastfed (yes or no) [20].

#### 2.2.4. Physical Activity and Diet

Markers of children’s physical activity were determined by questions related to participation in sport or exercise(yes or no), as well as average time spent daily watching television or playing on the computer. Children’s diets were measured by frequency of snacks (none, sometimes and often) [3].

#### 2.2.5. Family Socioeconomic Status

Family income per month was categorized into groups: (1) below ¥6000; (2) ¥6001–12,000; (3) ¥12,001–18,000; and (4) above ¥18,000. Parents’ education was classified into the following categories: (1) High school education or lower; (2) Associate degree; (3) Bachelor’s degree; (4) Master’s degree or above. Additionally, we included parents’ care for children with the question: “I am satisfied with the amount of time my parent and I spend together” [21,22], on a scale of 1 (strongly disagree) to 4 (strongly agree).

#### 2.2.6. School Environment

The school environment’s impact on weight was assessed by examining the amount of time students spent in sedentary or physical activities and eating habits during attendance. Students received physical education classes from one to four times a week. The number of physical education classes was compared to the number of other classes (1: below 5, 2: 5–7, 3: 8–10). Students also were asked whether or not they frequently go to the school store that sold food (yes or no). Because students were required to lunch at school, we measured time permitted for their meal (1: 1–15 min, 2: 16–30 min, 3: 31–40 min, 4: >40 min) [12].

### 2.3. Statistical Methods

All statistics were performed using SAS 9.0 (SAS Institute Inc., Cary, NC, USA). Descriptive analysis was used to describe participants’ characteristics and factors that possibly influenced outcomes. Chi-square tests and the t test were used to analyse the differences among characteristics between the public and private school students. To determine the association between overweight and possible factors in the two types of schools, we used multinomial logistic regression with adjustment for possible confounding variables.

### 2.4. Ethical Statement

All procedures performed in studies involving human participants were in accordance with the ethical standards of the institutional and/or national research committee and with the 1964 Helsinki declaration and its later amendments or comparable ethical standards. Procedures were approved by the ethical committee at Tongji University (LL-2014-ZRKX-012). Informed consent for measurements was sought from all eligible students. Written and informed consent was obtained from the students and the parents who participated in the study.

## 3. Results

### 3.1. Participant Characteristics

The demographics of the respondents are presented in Table 1. In our study, the overweight rate in private schools (15.20%) was significantly higher than in the public schools (10.18%, *p* = 0.021). Overall, male-to-female ratio was 1.17, and the average age of the respondents was 13.07 (SD = 0.89). A minority of the students were born overweight (7.98%) with a post-term pregnancy of 42 weeks and above (24.16%). Of them, 86.76% were breastfed during infancy. Regarding their physical activity, 58.09% answered with “often participate in physical activities each week”. Students spent 1.04 h per day on average watching television or playing computer games. The distributions of snacking frequencies (rarely, sometimes and often) were similar. The families of the public school students generally had higher incomes, as well as higher levels of paternal and maternal education than the private students. All students appraised the care they receive from their parents highly (Mean = 3.13, SD = 0.90). Most students responded that they took three or more physical education classes per week (68.38%). Only 34.14% of the students noted that they were allowed thirty minutes or more to eat their lunch. Data showed that private students had more classes every day (73.40% of them attended 8–10 classes per day). The number of students who often go to school stores was lower in the private schools (*p* < 0.001).

### 3.2. A Comparison of the Characteristics of Overweight Adolescents in Public and Private Schools

Table 2 shows the characteristics of overweight adolescents in public and private schools. The results indicated that differences with regard to excess weight between male and female students was larger in public schools (7.96%/2.21%) than in private ones (9.80%/5.40%). Students from private schools showed higher rates of excess weight compared to their public school counterparts.

Several factors contributed to a greater risk of excess weight amongst these students. Private school students born overweight demonstrated excessive weight at a rate of 1.60% compared to the 1.11% of the public school students. In particular, they exhibited a greater tendency toward being overweight if they were born overweight (1.60% compared to 1.11% of the private school students). Private students with mothers whose pregnancies lasted ≥42 weeks (6.20% compared to 3.76%) were more likely to be overweight than public students. Private students who were not breastfed during infancy (5.80% compared to 3.10%) had a higher ratio of being overweight. The rates of overweight students in public school who did not often engage in weekly exercise was 7.08% compared to 6.60% of the private school students. Public school overweight students spent less time watching television or playing computer games (public/private, mean: 1.18%/1.24%). Less public school overweight students often consumed daily snacks (public/private: 5.53%/9.20%).

Excess weight also correlated with family income. More students who were overweight were found among families that earned more than ¥6000 (below ¥6000: 1.37%, ¥6001–12,000: 7.14%, ¥12,001–18,000: 1.79%, above ¥18,000: 2.52%); excess weight among private students from families with earnings between ¥6001–12,000 interval proved particularly high (10.00%). The private school students who had parents with low levels of education exhibited greater rates of excess weight. However, public school students who had parents with low levels of education did not show higher rates of excess weight. Parental care also correlated with students’ weights in both private and public schools.

In terms of the school environment, the overweight rate was lower among all students who had more physical education classes per week and less other classes per day. For students often attending schools stores, the rate of excessive weight was higher among private school students (13.60%) than among public school students (8.63%). Students who had more than forty minutes for lunch demonstrated lower rates of excessive weigh (private/public: 0.80%/0.22%).

### 3.3. Analysis of Differences in Adolescent Weight

Logistic regression was used to analyse the differences in weight among adolescents (see Table 3). The results indicated that female students in public school showed a significantly lower risk of being overweight than male students (OR = 0.13, 95% CI: 0.04–0.37, *p* = 0.0002). The differences between females and males was less apparent among private school students (OR = 0.44, 95% CI: 0.20–0.98, *p* = 0.045). Public school students who were breastfed during infancy had significantly lower risk of being overweight compared to those who were not (OR = 0.27, 95% CI: 0.09–0.82, *p* = 0.021). Among the private school students, the correlation between breastfeeding and healthy weight proved even more striking (OR = 0.18, 95% CI: 0.09–0.39, *p* < 0.0001). In addition, results showed that private school students who were born to mothers who had a post-term pregnancy were at higher risk (OR = 4.50, 95% CI: 2.14–9.45, *p* < 0.0001) of being overweight. Yet, this was not the case for the public school students.

In contrast to the public school students, the private school students who sometimes ate snacks had a significantly lower risk of being overweight than those who frequently snacked (OR = 0.53, 95% CI: 0.24–1.16, *p* = 0.008). Surprisingly, only the private school students with family incomes in the ¥6001–12,000 range were more likely to be overweight than those from families who earned less than ¥6000 (OR = 3.27, 95% CI: 1.04–10.30, *p* = 0.043). Parental care was a very important factor in normal body weight for students from both types of schools (*p* < 0.0001).

The school environment played a more important role in the public school students’ weight; both the number of classes per day and time for lunch were found to be significant. Compared with students who took eight to ten classes per day in the public school group, those who responded that they had less than five showed a significantly lower risk for excess weight (OR = 0.02, 95% CI: 0.002–0.11, *p* < 0.0001). Among the private school students, the amount of classes taken was less significant (*p* = 0.015). Additionally, there was significant evidence that public school students who spent more than forty minutes on lunch (OR = 0.05, 95% CI: 0.003–0.82, *p* = 0.036) or 16–30 min (OR = 0.30, 95% CI: 0.09–0.98, *p* = 0.047) were less likely to be overweight. These findings differed significantly from the results among private school students.

In addition, the R^2^ change also indicated that, in total, personal characteristics and socioeconomic factors influenced the public school students more than the private school students. It seemed that factors related to early infancy, physical activity and diet, and school environment explained more of private school students’ obesity than public school students’.

## 4. Discussion

### 4.1. Rapid Immigration to Cities in China Proved Relevant the Increase in Adolescent Overweight

The rise in adolescent overweight has garnered much attention in recent years. A growing body of research on the topic has centered around a frequently discussed and uneven distribution of overweight across ethnicities in many countries [3,8,23]. However, our study identified another factor that contributes to overweight adolescents in Chinese cities like Shanghai with high rates of immigration from other less-developed areas; a system that limits access to equal educational resources. The rapid urbanization of some parts of China has contributed to this situation as registration in good public schools has become increasingly unavailable to large sectors of the population. The study showed that there exist significant disparities in the prevalence of excessive weight among adolescents in public and private schools in Shanghai. Students in top ranking public schools exhibited less likelihood of being overweight than those in the top private schools. From the early 2000s, local governments in large immigrant cities like Shanghai, Beijing and Guangzhou in China, strove to reduce the pressure of immigration on the school system through policies that restrict access to public education. In this case, only those students whose parents enjoyed registered residence in the city may be accepted by good public schools [16]. Therefore, it was understandable that differences in the personal characteristics, factors related to early infancy, physical activity and diet, socioeconomic factors, and school environment may have contributed to the discrepancy that existed in the prevalence of overweight adolescents in the two types of schools.

Determinant factors that could explain these disparities proved complex. They also differed in the two types of schools. The relationship between school type and adolescent overweight was complex because the observed disparities may have been related to factors that both preceded and followed school entry. This study examined a broad range of possible determinants. The results indicated that the differences between students in public and private schools with regard to overweight may be explained by factors related to early infancy, diet, socioeconomic status, and school environment.

Specifically, compared with their public school counterparts, the private school students were impacted more by early infancy-related and diet factors such as gestational age, breastfeeding, and snacking behavior. In their review, Lefebvre and John (2014) found that the majority of studies identified a relationship between breastfeeding and overweight prevention, but due to confounding maternal, child, cultural, genetic, and environmental variables, the relationship remained unclear [24]. This research has suggested that adolescents in private schools may have had parents whose infant-bearing knowledge was not sufficient. Studies have shown that parental feeding practices influence adolescents’ consumption of snacks [25,26]. It can be seen that parental care was both highly and positively associated with students’ maintain a healthy weight. This finding sustains Haines and colleagues’ argument (2016) that parents of students at different types of schools all paid great attention to their children’s growth and development [22].

In contradistinction to other studies that argued that lower socioeconomic status contributed to childhood overweight and obesity in western developed countries [27], this study has demonstrated that adolescents in private school from more affluent families (with a monthly income of between ¥6001–12,000) were more likely to be overweight than those whose families earned ¥6000 or less. Interestingly however, there was no observed difference among the wealthier families; this contrasts with findings from previous studies on other developing region [28]. 

The findings on public school students reaffirmed those evidenced in a preliminary study conducted fifteen years ago in Hong Kong; there was no correlation between childhood overweight/obesity and a set of socioeconomic parameters, such as parental education level and family income [29]. However, private school students whose family income was ¥6001–12,000 were more likely to be overweight. To date, an association between childhood overweight and socioeconomic factors remains unclear. The difference in public and private schools may be explained by the possibility that private students who live in middle-class families may receive less education about health from their parents.

In recent years, considerable attention has been given to the role schools play in the prevention of childhood overweight; children spend a large amount of their waking hours at school and it is there that they consume a substantial share of their daily calories [12]. For example, there is evidence to suggest that school policies attentive to food and physical activity are associated with improved obesogenic behaviors [30]. This study revealed that the environment for physical activities failed to explain the disparities identified, given that neither group of students showed weight variation in correlation with that variable. However, the number of classes and time provided for eating lunch could begin to explain disparities in overweight. In our study, students in public school who take more classes—thereby spending greater amounts of time in sedentary activities—evidenced a higher risk for excessive weight. Also, this study showed that public school students who are granted a greater amount of time to eat lunch have a lower risk of being overweight, in contrast to an earlier study on this same topic [12]. The greater pressure to perform academically experienced by students in good public schools could explain this difference. Those students may be more sensitive to concerns such as class time and time for lunch than private school students.

### 4.2. A Variety of Targeted Interventions Could Be Initiated for Students in the Two Types of Schools

The overweight situation identified, as well as the observed contributing factors, suggests an opportunity to address disparities in BMI in public and private schools. The Chinese government could devote more attention to the recruitment system that emerged from registration policies and that has resulted in unequal access to educational resources. Specific interventions could also be designed to address the particular concerns raised by this study. Because private school students are more sensitive to factors prior to school entry that contributed to overweight, such as early infancy factors, unhealthy food consumption and family income, it is essential to improve parents’ understandings about prenatal care as well as students’ and parents’ understanding about nutrition knowledge [3]. Interventions focused on breastfeeding, feeding patterns, parenting approach, active lifestyle, and other factors have shown promising results [19,31,32]. Given that the school environment risk factors explained a substantial amount of overweight for public school students, it would make sense to make adjustments to the number of classes and time provided for lunch.

### 4.3. Limitations

There are several limitations to this study worth noting. First, as the sample was chosen from several schools in Shanghai, China, the survey may not be representative of other immigrant cities. The survey should be extended to include a larger sample of Chinese schools in big cities with rapid immigration. Second, the data collected was based on self-reporting, which may produce bias due to the limitations of a participant’s ability to recall and/or report. Additionally, the only description of the school environment was based on data related to physical education classes and diet. Possible factors that could also be relevant would include considerations such as individual teachers’ education regarding healthy activities and diet.

## 5. Conclusions

Despite the limitations, the present study provides good evidence to illustrate health disparities in adolescent overweight between public and private school students in cities with high rates of immigration. Further research on individual, family and school environmental factors should be conducted to determine the impact on population health and to reduce the inequities that exist between public and private school students. This study has taken an important step in this direction and identified some potential avenues for future research.

## Figures and Tables

**Table 1:** Description of the respondents (*n* = 952), *n* (%).

Variable		Public School	Private School	Total	*p* Value
		452 (47.48%)	500 (52.52%)	952 (100%)	
Weight status	Not overweight	406 (89.82)	424 (84.80)	830 (87.18)	0.021
	Overweight	46 (10.18)	76 (15.20)	122 (12.82)	
*Personal characteristics*					
Gender	Male	242 (53.54)	272 (54.40)	514 (53.99)	0.790
	Female	210 (46.46)	228 (45.60)	438 (46.01)	
Age, mean (SD)		13.05 (0.92)	13.09 (0.87)	13.07 (0.89)	0.517
Factors related to early infancy					
Overweight at birth	No	417 (92.26)	459 (91.80)	876 (92.02)	0.795
	Yes	35 (7.74)	41 (8.20)	76 (7.98)	
Gestational age ≥42 weeks	No	340 (75.22)	382 (76.40)	722 (75.84)	0.672
	Yes	112 (24.78)	118 (23.60)	230 (24.16)	
Breastfeeding	No	49 10.84)	77 (15.40)	126 (13.24)	0.038
	Yes	403 (89.16)	423 (84.60)	826 (86.76)	
*Physical activity and diet*					
Weekly exercise	Not often	229 (50.66)	170 (34.00)	399 (41.91)	<0.0001
	Often	223 (49.34)	330 (66.00)	553 (58.09)	
Time spent watching television or playing computer games, mean (SD)		1.01 (1.39)	1.06 (1.52)	1.04 (1.46)	0.651
Number of snacks per day	Rarely	144 (31.86)	155 (31.00)	299 (31.41)	0.849
	Sometimes	144 (31.86)	168 (33.60)	312 (32.77)	
	Often	164 (36.28)	177 (35.40)	341 (35.82)	
*Socioeconomic factors*					
Family monthly income (¥)	Below 6000	26 (5.75)	76 (15.20)	102 (10.71)	<0.0001
	6001–12,000	157 (34.73)	242 (48.40)	399 (41.91)	
	12,001–18,000	110 (24.34)	89 (17.80)	199 (20.90)	
	Over 18,000	159 (35.18)	93 (18.60)	252 (26.47)	
Paternal education	High school degree or below	83 (18.36)	221 (44.20)	304 (31.93)	<0.0001
	Associate’s degree	111 (24.56)	160 (32.00)	271 (28.47)	
	Bachelor’s degree	134 (29.65)	85 (17.00)	219 (23.00)	
	Master’s degree or higher	124 (27.43)	34 (6.80)	158 (16.60)	
Maternal education	High school degree or below	100 (22.12)	177 (35.40)	277 (29.10)	<0.0001
	Associate degree	92 (20.35)	196 (39.20)	288 (30.25)	
	Bachelor’s degree	133 (29.42)	84 (16.80)	217 (22.79)	
	Master’s degree or higher	127 (28.10)	43 (8.60)	170 (17.86)	
Care from the parents, mean (SD)		3.15 (0.87)	3.12 (0.93)	3.13 (0.90)	0.632
*School environment*					
Number of physical education classes per week	0	28 (6.19)	50 (10.00)	78 (8.19)	0.057
	1–2	108 (23.89)	115 (23.00)	223 (23.42)	
	3–4	159 (35.18)	146 (29.20)	305 (32.04)	
	Over 4	157 (34.73)	189 (37.80)	346 (36.34)	
Number of classes per day, excluding physical education	Below 5	62 (13.72)	33 (6.60)	95 (9.98)	<0.0001
	5–7	131 (28.98)	100 (20.00)	231 (24.26)	
	8–10	259 (57.30)	367 (73.40)	626 (65.76)	
Often go to school stores that sell snacks	No	26 (5.75)	68 (13.60)	94 (9.87)	<0.0001
	Yes	426 (94.25)	432 (86.40)	858 (90.13)	
Duration of lunch period (minutes)	1–15	131 (28.98)	146 (29.20)	277 (29.10)	0.824
	16–30	172 (38.05)	178 (35.60)	350 (36.76)	
	31–40	121 (26.77)	146 (29.20)	267 (28.05)	
	Above 40	28 (6.20)	30 (6.00)	58 (6.09)	

**Table 2:** Distribution of overweight students and possible determining factors in two types of schools.

Variable		Public School *n* = 452		Private School *n* = 500		Total *n* = 952	
		None	Overweight	None	Overweight	None	Overweight
		406 (89.82%)	46 (10.18%)	424 (84.80%)	76 (15.20%)	830 (87.18%)	122 (12.82%)
Personal characteristics							
Gender	Male	206 (45.58)	36 (7.96)	223 (44.60)	49 (9.80)	429 (45.06)	85 (8.93)
	Female	200 (44.25)	10 (2.21)	201 (40.20)	27 (5.40)	401 (42.12)	37 (3.89)
Age	Mean (SD)	13.06 (0.92)	13.05 (0.92)	13.07 (0.88)	13.22 (0.81)	13.06 (0.90)	13.16 (0.86)
*Factors related to early infancy*							
Overweight at birth	No	376 (83.19)	41 (9.07)	391 (78.20)	68 (13.60)	767 (80.57)	109 (11.45)
	Yes	30 (6.64)	5 (1.11)	33 (6.60)	8 (1.60)	63 (6.62)	13 (1.37)
Gestational age ≥ 42 weeks	No	311 (68.81)	29 (6.42)	337 (67.40)	45 (9.00)	648 (68.07)	74 (7.77)
	Yes	95 (21.02)	17 (3.76)	87 (17.40)	31 (6.20)	182 (19.12)	48 (5.04)
Breastfeeding	No	35 (7.74)	14 (3.10)	48 (9.60)	29 (5.80)	83 (8.72)	43 (4.52)
	Yes	371 (82.08)	32 (7.08)	376 (75.20)	47 (9.40)	747 (78.47)	79 (8.30)
*Physical activity and diet*							
Weekly exercise	Not often	197 (43.58)	32 (7.08)	137 (27.40)	33 (6.60)	334 (35.08)	65 (6.83)
	Often	209 (46.24)	14 (3.10)	287 (57.40)	43 (8.60)	496 (52.10)	57 (5.99)
Time spent watching television or playing computer games, mean (SD)	Mean (SD)	1.00 (1.40)	1.18 (1.28)	1.02 (1.56)	1.24 (1.25)	1.01 (1.49)	1.22 (1.26)
Frequency of snacks per day	Rarely	129 (28.54)	15 (3.32)	137 (27.40)	18 (3.60)	266 (27.94)	33 (3.47)
	Sometimes	138 (30.53)	6 (1.33)	156 (31.20)	12 (2.40)	294 (30.88)	18 (1.89)
	Often	139 (30.75)	25 (5.53)	131 (26.20)	46 (9.20)	270 (28.36)	71 (7.46)
*Socioeconomic factors*							
Family monthly income (¥)	Below 6000	20 (4.42)	6 (1.33)	69 (13.80)	7 (1.40)	89 (9.35)	13 (1.37)
	6001–12,000	139 (30.75)	18 (3.98)	192 (38.40)	50 (10.00)	331 (34.77)	68 (7.14)
	12,001–18,000	101 (22.35)	9 (1.99)	81 (16.20)	8 (1.60)	182 (19.12)	17 (1.79)
	Over 18,000	146 (32.30)	13 (2.88)	82 (16.40)	11 (2.20)	228 (23.95)	24 (2.52)
Paternal education	High school degree or below	72 (15.93)	11 (2.43)	185 (37.00)	36 (7.20)	257 (27.00)	47 (4.94)
	Associate degree	101 (22.35)	10 (2.21)	141 (28.20)	19 (3.80)	242 (25.42)	29 (3.05)
	Bachelor’s degree	122 (26.99)	12 (2.65)	69 (13.80)	16 (3.20)	191 (20.06)	28 (2.94)
	Master’s degree or higher	111 (24.56)	13 (2.88)	29 (5.80)	5 (1.00)	140 (14.71)	18 (1.89)
Maternal education	High school degree or below	89 (19.69)	11 (2.43)	147 (29.40)	30 (6.00)	236 (24.79)	41 (4.31)
	Associate degree	78 (17.26)	14 (3.10)	172 (34.40)	24 (4.80)	250 (26.26)	38 (3.99)
	Bachelor’s degree	126 (27.88)	7 (1.55)	70 (14.00)	14 (2.80)	196 (20.59)	21 (2.21)
	Master’s degree or higher	113 (25.00)	14 (3.10)	35 (7.00)	8 (1.60)	148 (15.55)	22 (2.31)
Parental care, mean (SD)		3.24 (0.83)	2.30 (0.81)	3.26 (0.88)	2.30 (0.77)	3.25 (0.85)	2.30 (0.78)
*School environment*							
Number of physical education classes per week	0	26 (5.75)	2 (0.44)	38 (7.60)	12 (2.40)	64 (6.72)	14 (1.47)
	1–2	96 (21.24)	12 (2.65)	100 (20.00)	15 (3.00)	196 (20.59)	27 (2.84)
	3–4	147 (32.52)	12 (2.65)	124 (24.80)	22 (4.40)	271 (28.47)	34 (3.57)
	Over 4	137 (30.31)	20 (4.42)	162 (32.40)	27 (5.40)	299 (31.41)	47 (4.94)
Number of classes per day, excluding physical education	Below 5	59 (13.05)	3 (0.66)	29 (5.80)	4 (0.80)	88 (9.24)	7 (0.74)
	5–7	116 (25.66)	15 (3.32)	85 (17.00)	15 (3.00)	201 (21.11)	30 (3.15)
	8–10	231 (51.11)	28 (6.19)	310 (62.00)	57 (11.40)	541 (56.83)	85 (8.93)
Often go to school stores selling snacks	No	19 (4.20)	7 (1.55)	60 (12.00)	8 (1.60)	79 (8.30)	15 (1.58)
	Yes	387 (85.62)	39 (8.63)	364 (72.80)	68 (13.60)	751 (78.89)	107 (11.24)
Duration of lunch period (min)	1–15	115 (25.44)	16 (3.54)	125 (25.00)	21 (4.20)	240 (25.21)	37 (3.89)
	16–30	158 (34.96)	14 (3.10)	146 (29.20)	32 (6.40)	304 (31.93)	46 (4.83)
	31–40	106 (23.45)	15 (3.32)	127 (25.40)	19 (3.80)	233 (24.47)	34 (3.57)
	Over 40	27 (5.97)	1 (0.22)	26 (5.20)	4 (0.80)	53 (5.57)	5 (0.53)

**Table 3:** Logistic regression analysis of the differences among overweight adolescents in the two types of schools.

Variable		Public School ^#^			Private School ^§^		
		OR	95% CI	*p*	OR	95% CI	*p*
*Personal characteristics*							
Gender	Male	1.00	Reference		1.00	Reference	
	Female	0.13	0.04–0.37	0.0002	0.44	0.20–0.98	0.045
Age		1.11	0.64–1.92	0.708	1.06	0.70–1.59	0.790
*Factors related to early infancy*							
Overweight at birth	No	1.00	Reference		1.00	Reference	
	Yes	0.95	0.19–4.75	0.950	1.04	0.34–3.16	0.952
Gestational age ≥ 42 weeks	No	1.00	Reference		1.00	Reference	
	Yes	2.62	0.95–7.28	0.064	4.50	2.14–9.45	<0.0001
Breastfeeding	No	1.00	Reference		1.00	Reference	
	Yes	0.27	0.09–0.82	0.021	0.18	0.09–0.39	<0.0001
*Physical activity and diet*							
Exercise per week	No	1.00	Reference		1.00	Reference	
	Yes	0.40	0.15–1.03	0.059	0.52	0.23–1.15	0.105
Time spent watching television or playing computer games		1.09	0.85–1.40	0.489	0.97	0.80–1.17	0.740
Number of snacks per day	Rarely	2.12	0.72–6.27	0.174	0.30	0.12–0.73	0.113
	Sometimes	0.64	0.17–2.41	0.512	0.53	0.24–1.16	0.008
	Often	1.00	Reference		1.00	Reference	
*Socioeconomic factors*							
Family monthly income (¥)	Below 6000	1.00	Reference		1.00	Reference	
	6001–12,000	1.63	0.28–9.45	0.587	3.27	1.04–10.30	0.043
	12,001–18,000	0.47	0.06–3.54	0.460	1.06	0.25–4.63	0.934
	Over 18,000	1.17	0.16–8.41	0.877	1.26	0.32–4.99	0.746
Paternal education	High school degree or below	0.85	0.13–5.80	0.872	0.61	0.15–2.44	0.480
	Associate’s degree	0.89	0.23–3.44	0.868	0.52	0.13–2.14	0.369
	Bachelor’s degree	0.87	0.23–3.32	0.834	1.43	0.34–6.13	0.627
	Master’s degree or over	1.00	Reference		1.00	Reference	
Maternal education	High school degree or below	0.85	0.14–5.23	0.859	0.72	0.19–2.73	0.624
	Associate’s degree	1.27	0.39–4.13	0.692	0.99	0.28–3.49	0.983
	Bachelor’s degree	0.25	0.06–1.02	0.053	1.47	0.38–5.64	0.575
	Master’s degree or over	1.00	Reference		1.00	Reference	
Parental care		0.09	0.05–0.19	<0.0001	0.18	0.11–0.28	<0.0001
*School environment*							
Number of physical education classes per week	0	1.00	Reference		1.00	Reference	
	1–2	1.72	0.22–13.73	0.607	1.24	0.34–4.46	0.746
	3–4	1.29	0.16–10.20	0.808	2.00	0.60–6.68	0.257
	Over 4	5.00	0.60–41.61	0.137	2.09	0.63–6.94	0.229
Number of classes per day, excluding physical education	Below 5	0.02	0.002–0.11	<0.0001	0.16	0.04–0.70	0.015
	5–7	0.44	0.14–1.36	0.153	0.43	0.18–1.05	0.064
	8–10	1.00	Reference		1.00	Reference	
Often go to school stores selling snacks	No	1.00	Reference		1.00	Reference	
	Yes	0.37	0.07–1.95	0.241	1.45	0.51–4.13	0.483
Duration of lunch period (min)	1–15	1.00	Reference		1.00	Reference	
	16–30	0.30	0.09–0.98	0.047	0.66	0.28–1.55	0.341
	31–40	0.49	0.15–1.59	0.233	0.40	0.15–1.04	0.060
	Over 40	0.05	0.003–0.82	0.036	0.36	0.08–1.72	0.202

Notes: CI: Confidence interval; OR: Odds ratio; **^#^** R^2^ change in public school: the personal characteristics explained 11.94% of the obesity; factors related to early infancy explained 2.58% of the obesity; physical activity and diet explained 3.68% of the obesity; socioeconomic factors explained 12.94% of the obesity; school environment explained 11.46% of the obesity; **^§^** R^2^ change in private school: the personal characteristics explained 5.76% of the obesity; factors related to early infancy explained 12.12% of the obesity; physical activity and diet explained 6.98% of the obesity; socioeconomic factors explained 8.14% of the obesity; school environment explained 17.94% of the obesity.

## Availability of Data and Materials

Supplementary data related to this article can be found on line at https://figshare.com/s/2a8b79ec316478d4c5e8.
